# Supplementary material for: Investigating the clinico-anatomical dissociation in the behavioral variant of Alzheimer disease
Source: Alzheimers Res Ther. 2020 Nov 14;12:148. doi: 10.1186/s13195-020-00717-z (PMC7666520; doi:10.1186/s13195-020-00717-z)
Supplement: Supplementary file 1 — Additional file 1: : Supplement 1. Data availability per modality. [file 13195_2020_717_MOESM1_ESM.docx]

**Supplement 1 – Overview of availability of data per modality per patient group.**

| **Patient** | **T1** | **FLAIR** | **FDG** | **Patient** | **T1** | **FLAIR** | **FDG** | **Patient** | **T1** | **FLAIR** | **FDG** |
| --- | --- | --- | --- | --- | --- | --- | --- | --- | --- | --- | --- |
| bvAD51 | x | - | - | aAD01 | - | x | - | bvFTD111 | x | - | - |
| bvAD52 | x | - | - | aAD02 | - | x | - | bvFTD112 | x | - | - |
| bvAD53 | x | - | - | aAD03 | - | x | - | bvFTD113 | x | - | - |
| bvAD54 | x | - | - | aAD05 | - | x | - | bvFTD114 | x | - | - |
| bvAD55 | x | - | - | aAD06 | - | x | - | bvFTD115 | x | - | x |
| bvAD56 | x | - | - | aAD07 | - | x | - | bvFTD116 | x | - | - |
| bvAD57 | x | - | - | aAD11 | x | - | - | bvFTD117 | x | - | - |
| bvAD58 | x | - | x | aAD12 | x | - | x | bvFTD118 | x | - | - |
| bvAD59 | x | - | - | aAD13 | x | - | - | bvFTD119 | x | - | - |
| bvAD60 | x | - | - | aAD14 | x | - | - | bvFTD120 | x | - | - |
| bvAD61 | x | - | x | aAD15 | x | - | x | bvFTD121 | x | x | - |
| bvAD62 | x | - | - | aAD16 | x | - | x | bvFTD122 | x | x | - |
| bvAD64 | x | - | x | aAD17 | x | - | - | bvFTD123 | x | - | x |
| bvAD65 | x | x | x | aAD18 | x | - | - | bvFTD124 | x | x | - |
| bvAD67 | x | x | - | aAD19 | x | - | - | bvFTD125 | x | - | x |
| bvAD68 | x | x | - | aAD20 | x | - | x | bvFTD126 | x | x | x |
| bvAD69 | x | x | x | aAD21 | x | - | - | bvFTD127 | x | x | x |
| bvAD70 | x | x | x | aAD22 | x | x | x | bvFTD128 | x | x | x |
| bvAD73 | x | x | x | aAD23 | x | - | - | bvFTD129 | x | x | x |
| bvAD74 | x | x | x | aAD24 | x | - | - | bvFTD130 | x | x | x |
| bvAD75 | x | - | x | aAD25 | x | - | - | bvFTD131 | x | x | x |
| bvAD76 | x | x | - | aAD26 | x | - | - | bvFTD132 | x | x | x |
| bvAD77 | x | x | x | aAD27 | x | - | x | bvFTD133 | x | x | x |
| bvAD78 | x | x | x | aAD28 | x | - | x | bvFTD134 | x | x | x |
| bvAD79 | x | x | x | aAD29 | x | x | x | bvFTD135 | x | x | x |
| bvAD80 | x | x | x | aAD30 | x | - | x | bvFTD136 | x | x | x |
| bvAD81 | x | x | x | aAD31 | x | - | x | bvFTD137 | x | x | x |
| bvAD82 | x | x | x | aAD32 | x | - | x | bvFTD138 | x | x | x |
| bvAD83 | x | x | x | aAD33 | x | - | x | bvFTD139 | x | x | x |
| bvAD100 | - | - | x | aAD34 | x | x | x | bvFTD140 | x | x | x |
| bvAD101 | - | - | x | aAD35 | x | - | x |  |  |  |  |
| bvAD102 | - | - | x | aAD36 | x | x | x |  |  |  |  |
|  |  |  |  | aAD37 | x | - | x |  |  |  |  |
|  |  |  |  | aAD38 | x | x | x |  |  |  |  |
|  |  |  |  | aAD39 | x | x | x |  |  |  |  |
|  |  |  |  | aAD40 | - | x | - |  |  |  |  |

| **Controls** | **T1** | **FLAIR** | **FDG** | **Controls** | **T1** | **FLAIR** | **FDG** |
| --- | --- | --- | --- | --- | --- | --- | --- |
| HC100 | **-** | **-** | x | HC230 | x | x | - |
| HC101 | **-** | **-** | x | HC231 | x | x | - |
| HC102 | **-** | **-** | x | HC232 | x | - | - |
| HC103 | **-** | **-** | x | HC233 | x | x | - |
| HC104 | **-** | **-** | x | HC234 | x | - | - |
| HC105 | **-** | **-** | x | HC235 | x | x | - |
| HC106 | **-** | **-** | x | HC236 | x | x | - |
| HC107 | **-** | **-** | x | HC237 | x | x | - |
| HC108 | **-** | **-** | x | HC238 | x | - | - |
| HC109 | **-** | **-** | x | HC239 | x | - | - |
| HC110 | **-** | **-** | x | HC240 | x | - | - |
| HC111 | **-** | **-** | x | HC241 | x | - | - |
| HC112 | **-** | **-** | x | HC242 | x | - | - |
| HC113 | **-** | **-** | x | HC243 | x | - | - |
| HC114 | **-** | **-** | x | HC244 | x | - | - |
| HC115 | **-** | **-** | x | HC245 | x | x | - |
| HC116 | **-** | **-** | x | HC246 | x | - | - |
| HC117 | **-** | **-** | x | HC247 | x | - | - |
| HC118 | **-** | **-** | x | HC248 | x | x | - |
| HC119 | **-** | **-** | x | HC249 | x | x | - |
| HC120 | **-** | **-** | x | HC250 | x | x | - |
| HC121 | **-** | **-** | x | HC251 | x | - | - |
| HC122 | **-** | **-** | x | HC252 | x | x | - |
| HC123 | **-** | **-** | x | HC253 | x | - | - |
| HC124 | **-** | **-** | x | HC254 | x | - | - |
| HC125 | **-** | **-** | x | HC255 | x | - | - |
| HC126 | **-** | **-** | x | HC256 | x | x | - |
| HC127 | **-** | **-** | x | HC257 | x | x | - |
| HC128 | **-** | **-** | x | HC258 | x | x | - |
| HC129 | **-** | **-** | x | HC259 | x | x | - |
| HC130 | **-** | **-** | x | HC260 | x | x | - |
|  |  |  |  | HC261 | x | x | - |
|  |  |  |  | HC262 | x | x | - |
|  |  |  |  | HC263 | x | x | - |
