# Supplementary material for: Investigating the clinico-anatomical dissociation in the behavioral variant of Alzheimer disease
Source: Alzheimers Res Ther. 2020 Nov 14;12:148. doi: 10.1186/s13195-020-00717-z (PMC7666520; doi:10.1186/s13195-020-00717-z)
Supplement: Supplementary file 2 — Additional file 2: : Supplement 2. Participant characteristics of subgroups. [file 13195_2020_717_MOESM2_ESM.docx]

**Supplement 2 – Participant characteristics of the subgroups with FDG-PET and FLAIR-MRI available.**

**FDG-PET subgroups**

|  | bvAD | tAD | bvFTD | CN_2_ | p-value |
| --- | --- | --- | --- | --- | --- |
| n | 19 | 18 | 18 | 31 |  |
| Age, y | 66.1 (7.4) | 64.7 (9.6) | 63.7 (5.7) | 65.5 (8.0) | 0.80 |
| Sex, no. male (%) | 11 (58) | 9 (50) | 14 (78) | 19 (61) | 0.82 |
| Education, y mean (SD) | 15.8 (2.4) | 16.2 (3.0) | 15.5 (3.4) | - | 0.76 |
| MMSE ^†^, mean (SD) | 21.8 (5.7) | 21.5 (5.7) | 21.8 (7.1) | - | 0.99 |
| APOEε4 positivity^a^,  no. of patients (%) | 7/13 (54) | 9/11 (82) | 3/18 (17) | - | <0.01 |
| Scanner type^b^ |  |  |  |  | <0.001 |
| ECAT | 15 (79) | 18 (100) | 13 (72) | 12 (39) |  |
| BIO | 4 (21) | 0 (0) | 5 (28) | 19 (61) |  |
| Memory domain z-score^c^ °, mean (SD) | -3.2 (1.5) | -4.0 (1.3) | -2.2 (1.7) | - | <0.01 |
| Executive domain z-score°, mean (SD) | -1.6 (1.0) | -1.8 (1.0) | -1.7 (1.0) | - | 0.88 |
| NPI score^d ◊^, mean (SD) | 31.8 (23.2) | 11.5 (13.5) | 37.2 (19.7) | **-** | <0.01 |

Differences between groups were assessed using ANOVA tests, Chi-square tests and Kruskall-Wallis tests with post hoc Mann-Whitney U-tests, where appropriate. All p-values were corrected for multiple comparisons using Bonferroni correction. ▪ MMSE data was available for n=13 for bvAD, n=13 for tAD, n=17 for bvFTD and n=0 for CN_2,_ ° Cognition data was available for n=15 for bvAD, n=18 for tAD, n=15 for bvFTD, n=0 for CN_2_ and assessed using a MANOVA with Bonferroni correction, ^◊^ NPI data was available for n=8 for bvAD, n=4 for tAD, n=10 for bvFTD and n=0 for CN_2,_ ^a^tAD & bvAD > bvFTD, p<0.05, ^b^tAD < bvFTD, p<0.01, ^c^Controls > patients, p<0.001, ^d^bvFTD > tAD, p<0.01.

|  | bvAD | tAD | bvFTD | CN_1_ | p-value |
| --- | --- | --- | --- | --- | --- |
| n | 15 | 14 | 18 | 19 |  |
| Age, y | 66.2 (8.8) | 61.6 (6.9) | 64.2 (5.1) | 67.2 (9.7) | 0.22 |
| Sex, no. male (%) | 8 (53) | 6 (43) | 13 (72) | 12 (63) | 0.37 |
| Education, y mean (SD) | 15.5 (2.8) | 16.8 (3.3) | 15.6 (3.4) | 17.2 (2.3) | 0.26 |
| MMSE^a^ ^†^, mean (SD) | 21.9 (5.8) | 20.4 (6.3) | 21.4 (7.3) | 29.3 (0.8) | <0.001 |
| APOEε4 positivity^b^,  no. of patients (%) | 9/13 (69) | 4/4 (100) | 3/18 (17) | 1/19 (5) | <0.001 |
| Scanner type |  |  |  |  | 0.33 |
| 1.5T | 4 (27) | 3 (21) | 2 (11) | 7 (37) |  |
| 3T | 11 (73) | 11 (79) | 16 (89) | 12 (63) |  |
| Memory domain z-score^c^ °, mean (SD) | -3.4 (1.6) | -4.1 (1.1) | -2.3 (1.8) | 0.3 (0.8) | <0.001 |
| Executive domain z-score^d^ °, mean (SD) | -2.0 (1.1) | -1.7 (1.1) | -1.8 (1.0) | -0.1 (0.6) | <0.001 |
| NPI score^◊^, mean (SD) | 31.8 (23.2) | 11.5 (13.5) | 37.2 (19.7) | **-** | 0.11 |

**MRI-FLAIR subgroups**

Differences between groups were assessed using ANOVA tests, Chi-square tests and Kruskall-Wallis tests with post hoc Mann-Whitney U-tests, where appropriate. All p-values were corrected for multiple comparisons using Bonferroni correction. ^†^ MMSE data was available for n=12 for bvAD, n=10 for tAD, n=17 for bvFTD and n=19 for CN_1_, ° Cognition data was available for n=15 for bvAD, n=6 for tAD, n=16 for bvFTD, n=17 for CN_1_ and assessed using a MANOVA with Bonferroni correction, ^◊^ NPI data was available for n=8 for bvAD, n=4 for tAD, n=10 for bvFTD and n=0 for CN_1_, ^a^Controls > patients, p<0.05, ^b^Controls < tAD & bvAD, p<0.001, tAD & bvAD > bvFTD, p<0.01, ^c^Controls > patients, p<0.001, ^d^Controls > bvAD & bvFTD, p<0.001, controls > tAD, p<0.01.
