# Supplementary material for: Investigating the clinico-anatomical dissociation in the behavioral variant of Alzheimer disease
Source: Alzheimers Res Ther. 2020 Nov 14;12:148. doi: 10.1186/s13195-020-00717-z (PMC7666520; doi:10.1186/s13195-020-00717-z)
Supplement: Supplementary file 3 — Additional file 3: : Supplement 3. Goodness-of-fit scores of networks. [file 13195_2020_717_MOESM3_ESM.docx]

**Supplement 3 – Goodness-of-fit (GOF) scores of networks compared to functional network templates.**

**Comparison with functional network templates by Shirer et al. (2012)**

|  | bvAD | tAD | bvFTD | CN_2_ |
| --- | --- | --- | --- | --- |
| pDMN | 3.85 | 4.14 | 2.04 | 1.38 |
| aDMN | 4.13 | 2.92 | 4.13 | 2.60 |
| SAL | 1.05 | 0.62 | 2.90 | 0.76 |
| ECN | 2.20 | 3.11 | 2.78 | 1.92 |

GOF scores represent the subtraction of the mean *T*-score outside of the network template from the mean *T*-score within the network template, and GOF ratios represent the division of the mean *T*-score inside the network template by the mean *T*-score outside the network template. *pDMN* = posterior default mode network; *aDMN* = anterior default mode network; *SAL* = salience network; *ECN* = executive control network.

|  | bvAD | tAD | bvFTD | CN_2_ |
| --- | --- | --- | --- | --- |
| pDMN | 1.49 | 1.27 | 0.77 | 0.88 |
| aDMN | 2.09 | 1.41 | 2.09 | 1.37 |
| SAL | 0.71 | 0.74 | 1.82 | 0.83 |
| ECN | 2.46 | 3.57 | 3.02 | 1.31 |

**Comparison with** **functional network templates by Neurosynth.org.**

GOF scores represent the subtraction of the mean *T*-score outside of the network template from the mean *T*-score within the network template, and GOF ratios represent the division of the mean *T*-score inside the network template by the mean *T*-score outside the network template. *pDMN* = posterior default mode network; *aDMN* = anterior default mode network; *SAL* = salience network; *ECN* = executive control network.

**
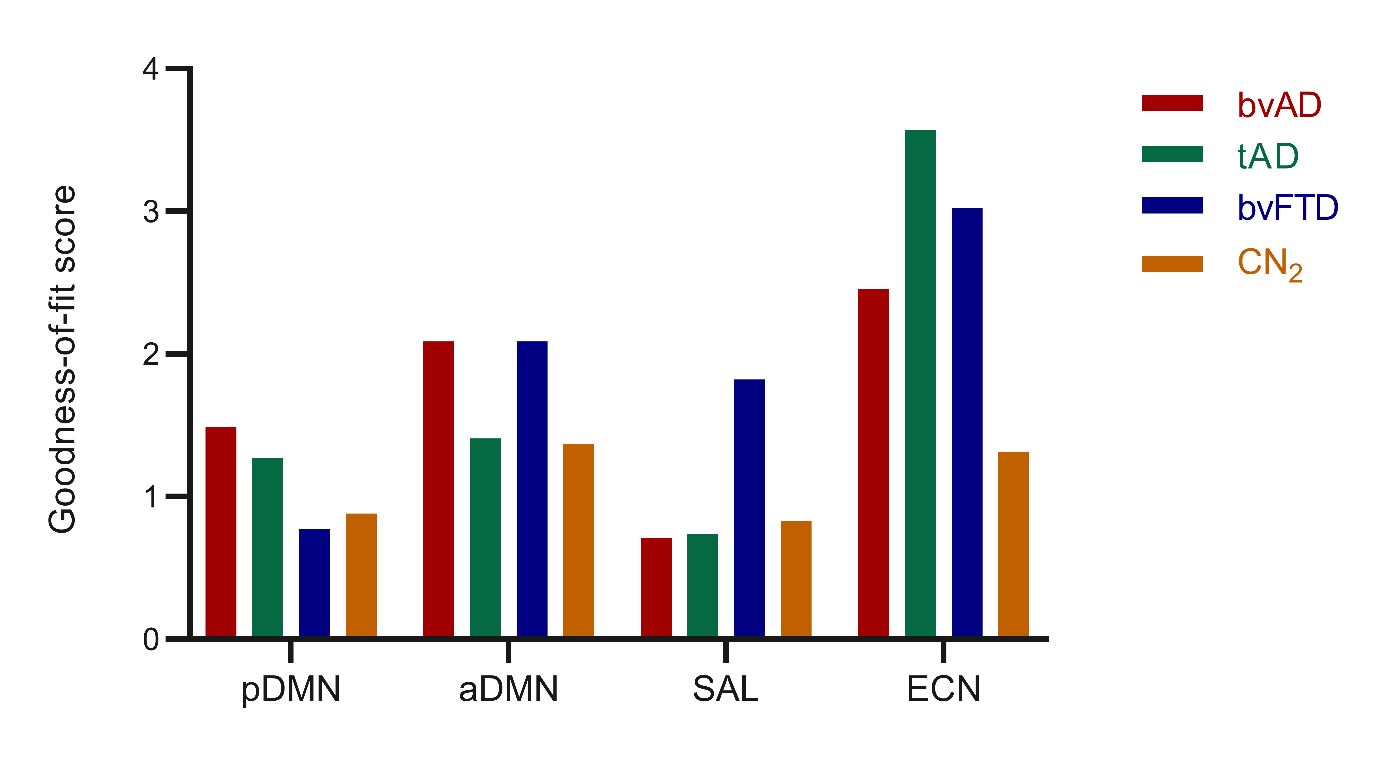
Goodness-of-fit (GOF) scores and ratios indicating the resemblance of the FDG interregional covariance maps with the functional network templates obtained from Neurosynth.org.**

GOF scores represent the subtraction of the mean *T*-score outside of the network template from the mean *T*-score within the network template, and GOF ratios represent the division of the mean *T*-score inside the network template by the mean *T*-score outside the network template. *pDMN* = posterior default mode network, *aDMN* = anterior default mode network, *SAL* = salience network, *ECN* = executive control network

**
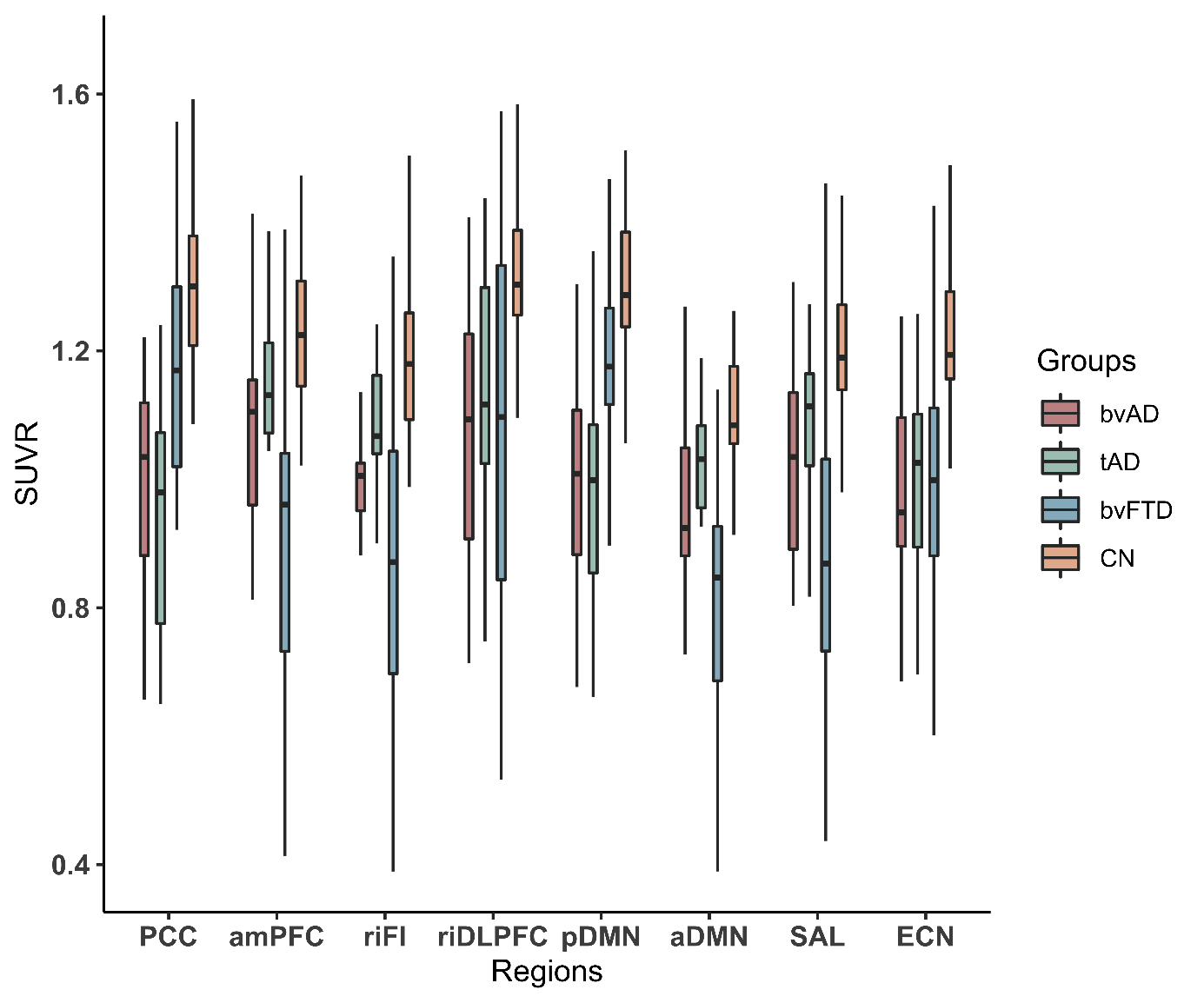
Metabolism in seed regions and network templates**
