# Supplementary material for: Investigating the clinico-anatomical dissociation in the behavioral variant of Alzheimer disease
Source: Alzheimers Res Ther. 2020 Nov 14;12:148. doi: 10.1186/s13195-020-00717-z (PMC7666520; doi:10.1186/s13195-020-00717-z)
Supplement: Supplementary file 4 — Additional file 4: : Supplement 4. Clusters in SPM interaction analysis. [file 13195_2020_717_MOESM4_ESM.docx]

**Supplement 4 – Information on most significant clusters in patients vs patients contrasts in the SPM interaction analysis of metabolic connectivity**

| Seed | Contrast set at p_unc_ <0.001, k=0 extent threshold | Cluster equiv k | Cluster p-value | Peak T-value | Peak equivalent z value | Peak p-value (uncorrected) | X | Y | Z |
| --- | --- | --- | --- | --- | --- | --- | --- | --- | --- |
| PCC | bvAD > tAD | 15 | 0.744 | 3.45 | 3.32 | 0.000 | -40 | -92 | -6 |
|  | bvAD < tAD | 14 | 0.754 | 3.39 | 3.26 | 0.001 | 38 | 14 | 24 |
|  | bvAD > bvFTD | 23118 | 0.000 | 5.34 | 4.92 | 0.000 | -2 | 32 | 18 |
|  | bvAD < bvFTD | 300 | 0.113 | 3.93 | 3.74 | 0.000 | 44 | -58 | 28 |
|  | tAD > bvFTD | 34651 | 0.000 | 6.70 | 5.95 | 0.000 | 34 | 64 | -4 |
|  | tAD < bvFTD | 169 | 0.226 | 5.47 | 5.02 | 0.000 | -12 | -104 | -10 |
| riFI | bvAD > tAD | 181 | 0.190 | 3.76 | 3.59 | 0.000 | -30 | -6 | -34 |
|  | bvAD < tAD | 3 | 0.898 | 3.34 | 3.22 | 0.001 | 8 | 6 | -4 |
|  | bvAD > bvFTD | 5 | 0.859 | 3.50 | 3.37 | 0.000 | -44 | -24 | 36 |
|  | bvAD < bvFTD | 30317 | 0.000 | 9.56 | 7.76 | 0.000 | 46 | -62 | 32 |
|  | tAD > bvFTD | 91 | 0.349 | 3.81 | 3.64 | 0.000 | 40 | 16 | -14 |
|  | tAD < bvFTD | 37739 | 0.000 | 3.22 | 3.11 | 0.001 | -2 | -96 | 26 |

*PCC* = posterior cingulate cortex, *riFI* = right frontoinsula.
