# Supplementary material for: Investigating the clinico-anatomical dissociation in the behavioral variant of Alzheimer disease
Source: Alzheimers Res Ther. 2020 Nov 14;12:148. doi: 10.1186/s13195-020-00717-z (PMC7666520; doi:10.1186/s13195-020-00717-z)
Supplement: Supplementary file 5 — Additional file 5: : Supplement 5. Subcortical and white matter volume. [file 13195_2020_717_MOESM5_ESM.docx]

**Supplement 5 – Deep grey matter volumes and regional white matter hyperintensities across groups.**

**Deep grey matter volumes of subcortical structures across groups, presented as mean volume in cm^3^.**

|  | bvAD | tAD | bvFTD | CN_1_ |
| --- | --- | --- | --- | --- |
| n | 29 | 28 | 28 | 34 |
| Hippocamus^a^ | 5.85 (1.10) | 5.45 (0.76) | 5.49 (1.09) | 6.55 (0.87) |
| Amygdala^b^ | 2.73 (0.39) | 2.33 (0.46) | 2.45 (0.66) | 2.82 (0.40) |
| Nucleus Accumbens^c^ | 0.72 (0.22) | 0.73 (0.21) | 0.51 (0.18) | 0.91 (0.21) |
| Caudate nucleus^d^ | 6.11 (0.86) | 6.07 (0.59) | 5.70 (0.92) | 6.54 (0.70) |
| Pallidum^e^ | 3.48 (0.57) | 3.48 (0.43) | 3.12 (0.59) | 3.63 (0.33) |
| Putamen^f^ | 8.82 (1.50) | 8.89 (0.77) | 8.22 (1.34) | 9.70 (0.90) |
| Thalamus^g^ | 14.71 (1.82) | 14.37 (1.29) | 14.44 (1.74) | 15.85 (1.16) |

^a^ Controls > patients, *p*<0.001
^b^ Controls > tAD, p=0.001, controls > bvFTD, p<0.05, bvAD > tAD, p<0.05
^c^ Controls > tAD, p<0.05, controls > bvFTD, p<0.001, bvFTD < tAD, p<0.05
^d^ Controls > bvAD, p<0.05, controls > bvFTD, p<0.001
^e^ Controls > bvFTD, p<0.001, bvFTD < bvAD & tAD, p<0.05
^f^ Controls > bvFTD, p<0.001, controls > bvAD, p=0.01
^g^ Controls > bvAD, p<0.01, controls > tAD, p<0.05, controls > bvFTD, p<0.001

|  | bvAD | tAD | bvFTD | CN_1_ |
| --- | --- | --- | --- | --- |
| n | 15 | 14 | 18 | 19 |
| Frontal lobe | 1.26 (1.16) | 1.24 (2.35) | 2.11 (2.85) | 0.51 (0.66) |
| Parietal lobe | 0.51 (0.54) | 0.77 (1.28) | 0.81 (1.42) | 0.29 (0.65) |
| Occipital lobe | 0.50 (0.56) | 0.45 (0.73) | 0.37 (0.31) | 0.21 (0.35) |
| Temporal lobe | 0.25 (0.19) | 0.27 (0.31) | 0.48 (0.67) | 0.16 (0.31) |
| Basal Ganglia & Infratentorial region^a^ | 0.12 (0.14) | 0.11 (0.29) | 0.08 (0.09) | 0.02 (0.03) |

**Weighted regional white matter hyperintensity volumes, presented as mean volume in cm^3^ [95%CI].**

^a^ Controls < bvAD & bvFTD, p<0.01
