# Supplementary material for: Investigating the clinico-anatomical dissociation in the behavioral variant of Alzheimer disease
Source: Alzheimers Res Ther. 2020 Nov 14;12:148. doi: 10.1186/s13195-020-00717-z (PMC7666520; doi:10.1186/s13195-020-00717-z)

|  |  | bvAD | tAD | bvFTD | CN_1_ | CN_2_ |
| --- | --- | --- | --- | --- | --- | --- |
| T1-MRI^◊^ | n | 16 | 18 | 17 | 34 | - |
|  | Temporoparietal cortex^a^ | 0.37 (0.08) | 0.37 (0.07) | 0.42 (0.07) | 0.49 (0.04) | - |
|  | Total parietal cortex^b^ | 0.27 (0.06) | 0.26 (0.05) | 0.31 (0.04) | 0.34 (0.03) | - |
|  | Hippocampus^c^ | 0.32 (0.06) | 0.31 (0.05) | 0.33 (0.08) | 0.40 (0.03) | - |
|  | FTD signature region^d^ | 0.31 (0.06) | 0.32 (0.05) | 0.28 (0.07) | 0.38 (0.03) | - |
|  | Amygdala^e^ | 2.72 (0.33) | 2.21 (0.47) | 2.40 (0.70) | 2.82 (0.39) | - |
|  | Posterior DMN^f^ | 1.01 (0.10) | 1.00 (0.11) | 1.19 (0.11) | 1.10 (0.05) | - |
|  | Anterior DMN^g^ | 1.03 (0.08) | 1.11 (0.08) | 0.86 (0.16) | 1.05 (0.07) | - |
|  | Salience Network^h^ | 1.09 (0.07) | 1.12 (0.05) | 0.93 (0.12) | 1.08 (0.05) | - |
|  | Executive Control Network^i^ | 0.95 (0.07) | 0.98 (0.07) | 0.97 (0.07) | 1.01 (0.04) | - |
| FDG-PET^†^ | n | 19 | 18 | 18 | - | 31 |
|  | Temporoparietal cortex^j^ | 1.00 (0.13) | 0.99 (0.11) | 1.11 (0.14) | - | 1.22 (0.09) |
|  | Total parietal cortex^k^ | 0.96 (0.13) | 0.94 (0.16) | 1.10 (0.11) | - | 1.16 (0.10) |
|  | FTD signature region^l^ | 0.93 (0.11) | 0.97 (0.10) | 0.82 (0.21) | - | 1.05 (0.08) |

**Supplement 6 – Results of the Receiver-Operating-Curve analysis**

| FDG-PET | Posterior DMN^m^ | 1.00 (0.14) | 0.99 (0.13) | 1.18 (0.06) | - | 1.17 (0.04) |
| --- | --- | --- | --- | --- | --- | --- |
|  | Anterior DMN^n^ | 0.98 (0.10) | 1.04 (0.10) | 0.80 (0.16) | - | 0.97 (0.04) |
|  | Salience Network^o^ | 1.05 (0.09) | 1.09 (0.7) | 0.90 (0.16) | - | 1.07 (0.04) |
|  | Executive Control Network^p^ | 0.98 (0.09) | 0.99 (0.08) | 1.00 (0.11) | - | 1.08 (0.03) |
| FLAIR-MRI^□^ | n | 15 | 14 | 18 | 19 | - |
|  | WMH Frontal lobe | 1.24 (2.35) | 1.26 (1.16) | 2.11 (2.85) | 0.51 (0.66) | - |
|  | WMH Parietal lobe | 0.77 (1.28) | 0.51 (0.54) | 0.81 (1.42) | 0.29 (0.65) | - |
|  | WMH Occipital lobe | 0.45 (0.73) | 0.50 (0.56) | 0.37 (0.31) | 0.21 (0.35) | - |
|  | WMH Temporal lobe | 0.27 (0.31) | 0.25 (0.19) | 0.48 (0.67) | 0.16 (0.31) | - |
|  | WMH Basal Ganglia and Infratentorial regions^q^ | 0.11 (0.29) | 0.12 (0.14) | 0.08 (0.09) | 0.02 (0.03) | - |

^◊^ MRI data presented were analyzed with a MANCOVA with Bonferroni correction, correcting for age, sex, scanner field strength and total intracranial volume.
^†^ FDG data presented above were analyzed with MANCOVA with Bonferroni correction, correcting for age, sex and scanner type.
^□^ WMH data presented above are presented in cm^3^ and were tested with Kruskall-Wallis and post hoc Mann-Whitney U tests, with manual Bonferroni correction (p<0.01).
^a^Controls > patients, p<0.001, bvAD < bvFTD, p=0.01, ^b^Controls > patients, p<0.001, bvAD < bvFTD, p<0.01, ^c^Controls > patients, p<0.001, ^d^Controls > bvAD & bvFTD, p<0.001, Controls > tAD, p<0.05, ^e^Controls > tAD, p=0.001, controls > bvFTD, p<0.05, bvAD > tAD, p<0.05, ^f^Controls > bvAD & tAD, p<0.05, bvFTD > controls, p<0.05, bvFTD > bvAD & tAD, p<0.001, ^g^bvFTD < bvAD, tAD & controls, p<0.001, ^h^bvFTD < bvAD, tAD & controls, p<0.001, ^I^No significant differences, ^j^Controls > tAD & bvAD, p<0.001, controls > bvFTD, p<0.05 , tAD < bvFTD, p<0.05, ^k^Controls > tAD & bvAD, p<0.001, tAD < bvFTD, p<0.05, ^l^Controls > tAD & bvAD, p<0.05, controls > bvFTD, p<0.001, ^m^Controls > bvAD & tAD, p<0.001, bvAD & tAD < bvFTD, p<0.001, ^n^Controls > bvFTD, p<0.001, bvAD & tAD > bvFTD, p<0.001, ^o^Controls > bvFTD, p<0.001, bvAD & tAD > bvFTD, p<0.001, ^p^Controls > tAD & bvFTD, p<0.001, controls > bvAD, p<0.01, ^q^Controls < bvAD & bvFTD, p<0.01.

**Top-5 discriminating measures per contrast, with corresponding area under the curve, 95% confidence intervals and significance levels.**

|  | bvAD *vs*. bvFTD | |  |  | bvAD *vs*. tAD |  |  | tAD *vs.* bvFTD |  |  |
| --- | --- | --- | --- | --- | --- | --- | --- | --- | --- | --- |
|  | Measure | AUC (95% CI) | | p-value | Measure | AUC (95% CI) | p-value | Measure | AUC (95% CI) | p-value |
| 1 | MRI pDMN | 0.91 (0.81-1.00) | | <0.001 | MRI AMYG | 0.84 (0.71-0.98) | <0.001 | MRI aDMN | 0.96 (0.89-1.00) | <0.001 |
| 2 | FDG pDMN | 0.91 (0.80-1.00) | | <0.001 | MRI aDMN | 0.75 (0.59-0.92) | 0.01 | MRI SAL | 0.94 (0.87-1.00) | <0.001 |
| 3 | MRI SAL | 0.85 (0.72-0.98) | | <0.001 | FDG aDMN | 0.71 (0.54-0.89) | 0.03 | FDG pDMN | 0.92 (0.82-1.00) | <0.001 |
| 4 | FDG aDMN | 0.83 (0.70-0.97) | | <0.001 | FDG SAL | 0.66 (0.49-0.84) | 0.09 | FDG aDMN | 0.92 (0.82-1.00) | <0.001 |
| 5 | MRI aDMN | 0.80 (0.65-0.95) | | <0.001 | MRI SAL | 0.65 (0.45-0.85) | 0.13 | MRI pDMN | 0.90 (0.81-1.00) | <0.001 |
|  | bvAD *vs*. CN |  | |  | tAD *vs*. CN |  |  | bvFTD *vs*. CN |  |  |
|  | Measure | AUC (95% CI) | | p-value | Measure | AUC (95% CI) | p-value | Measure | AUC (95% CI) | p-value |
| 1 | FDG TPC | 0.93 (0.86-1.00) | | <0.001 | FDG TPC | 0.98 (0.94-1.00) | <0.001 | MRI FTD | 0.88 (0.78-0.98) | <0.001 |
| 2 | FDG PAR | 0.91 (0.83-1.00) | | <0.001 | MRI HIP | 0.94 (0.88-1.00) | <0.001 | MRI aDMN | 0.87 (0.76-0.97) | <0.001 |
| 3 | MRI TPC | 0.91 (0.79-1.00) | | <0.001 | MRI TPC | 0.92 (0.83-1.00) | <0.001 | FDG aDMN | 0.87 (0.76-0.97) | <0.001 |
| 4 | FDG pDMN | 0.90 (0.79-1.00) | | <0.001 | FDG PAR | 0.91 (0.81-1.00) | <0.001 | MRI SAL | 0.86 (0.74-0.98) | <0.001 |
| 5 | MRI PAR | 0.88 (0.75-1.00) | | <0.001 | FDG pDMN | 0.90 (0.79-1.00) | <0.001 | WMH BGIT | 0.86 (0.72-0.99) | <0.001 |

*FDG TPC* = temporoparietal cortex metabolism on FDG-PET; *FDG PAR* = parietal cortex metabolism on FDG-PET; *FDG pDMN* = glucose metabolism within the posterior default mode network, divided by the glucose metabolism outside the posterior default mode network on FDG-PET; *FDG aDMN* = glucose metabolism within the anterior default mode network, divided by the glucose metabolism outside the anterior default mode network on FDG-PET; *FDG SAL* = glucose metabolism within the salience network, divided by the glucose metabolism outside the salience network on FDG-PET; *MRI FTD* = FTD signature region gray matter volume on MRI, consisting of the anterior cingulate, frontoinsula, striatum and frontopolar regions. *MRI AMYG* = bilateral amygdala gray matter volume on MRI; *MRI HIP*  = gray matter volume of the hippocampus on MRI; *MRI TPC* = temporoparietal gray matter volume on MRI; *MRI PAR* = parietal cortex gray matter volume on MRI; *MRI pDMN* = gray matter volume within the posterior default mode network, divided by the gray matter volume outside the posterior default mode network on MRI; *MRI aDMN* = gray matter volume within the anterior default mode network, divided by the gray matter volume outside the anterior default mode network on MRI; *MRI SAL* = gray matter volume within the salience network, divided by the gray matter volume outside the salience network on MRI; *WMH BGIT* = white matter hyperintensity volume within the basal ganglia and infratentorial regions on FLAIR MRI.

**Top-5 discriminating measures per contrast including all individuals with MRI available, with corresponding area-under-the-curve and 95% confidence intervals.**


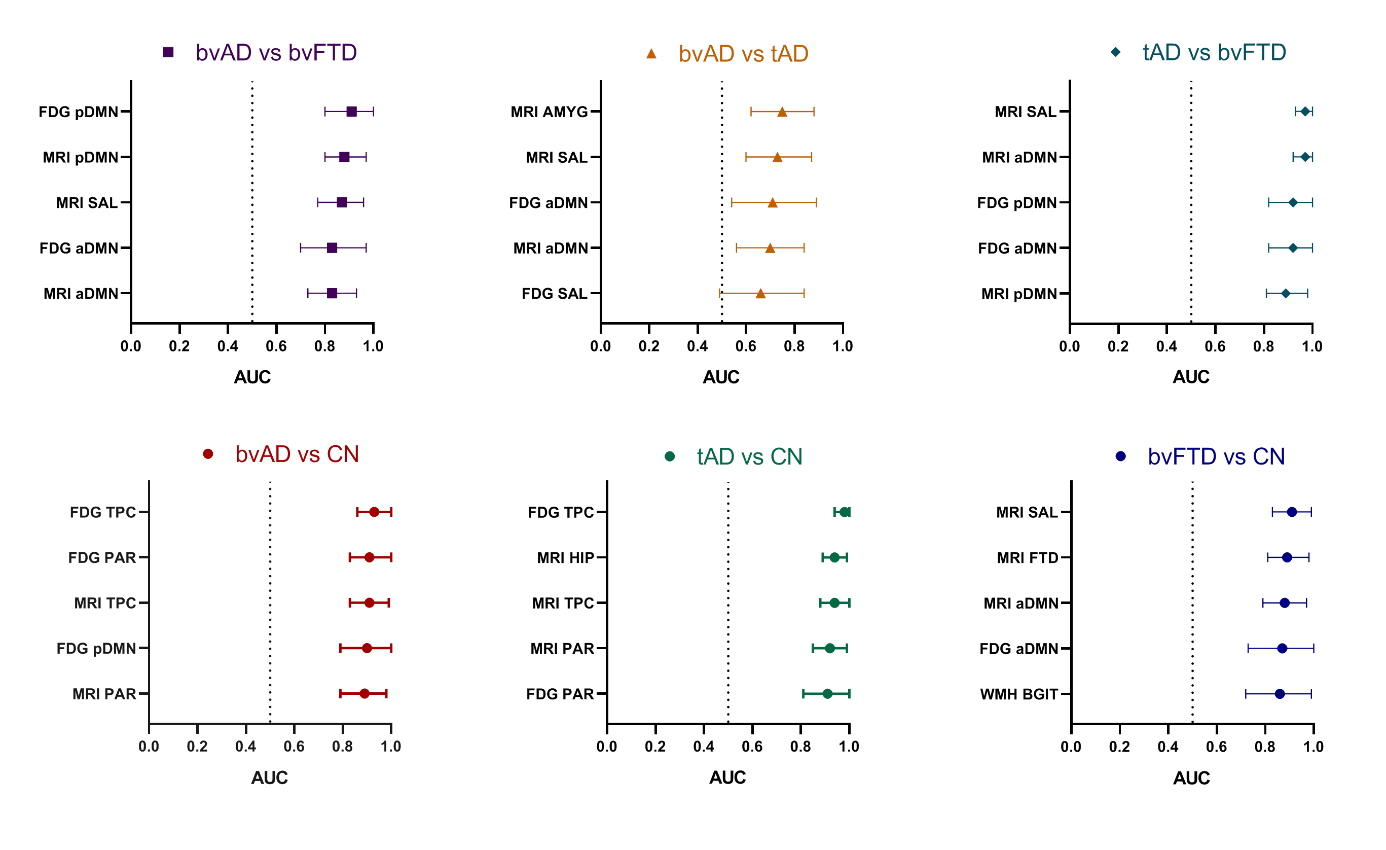

Supplement: Supplementary file 6 — Additional file 6: : Supplement 6. Receiver-operating-characteristics analysis. [file 13195_2020_717_MOESM6_ESM.docx]
