# Supplementary material for: Investigating the clinico-anatomical dissociation in the behavioral variant of Alzheimer disease
Source: Alzheimers Res Ther. 2020 Nov 14;12:148. doi: 10.1186/s13195-020-00717-z (PMC7666520; doi:10.1186/s13195-020-00717-z)
Supplement: Supplementary file 7 — Additional file 7: : Supplement 7. Hypometabolism between patients contrasts. [file 13195_2020_717_MOESM7_ESM.docx]

**Supplement 7 – Patterns of hypometabolism resulting from contrasts between patient groups.**

**
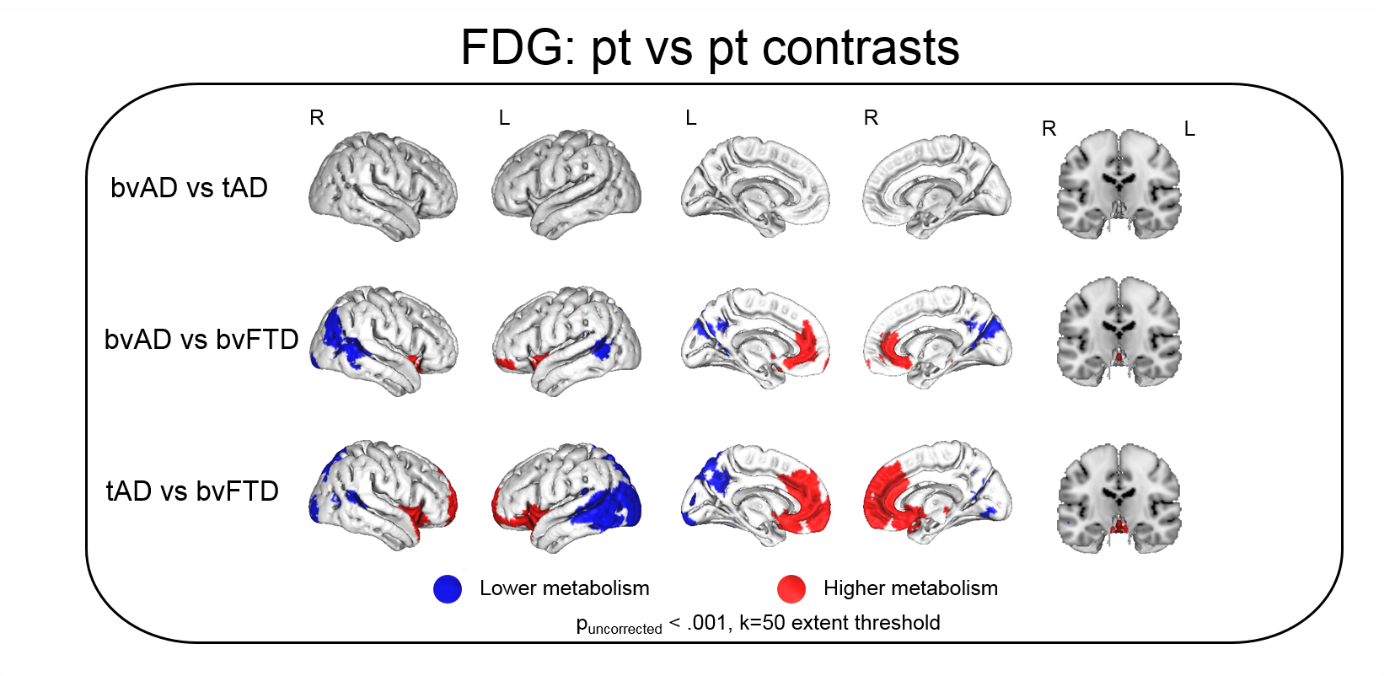
Patterns based on significant voxels.**

Surface rendering of significant voxels resulting from showing differences in metabolic in patients compared to other patient groups. Contrasts were adjusted for age and sex.

**
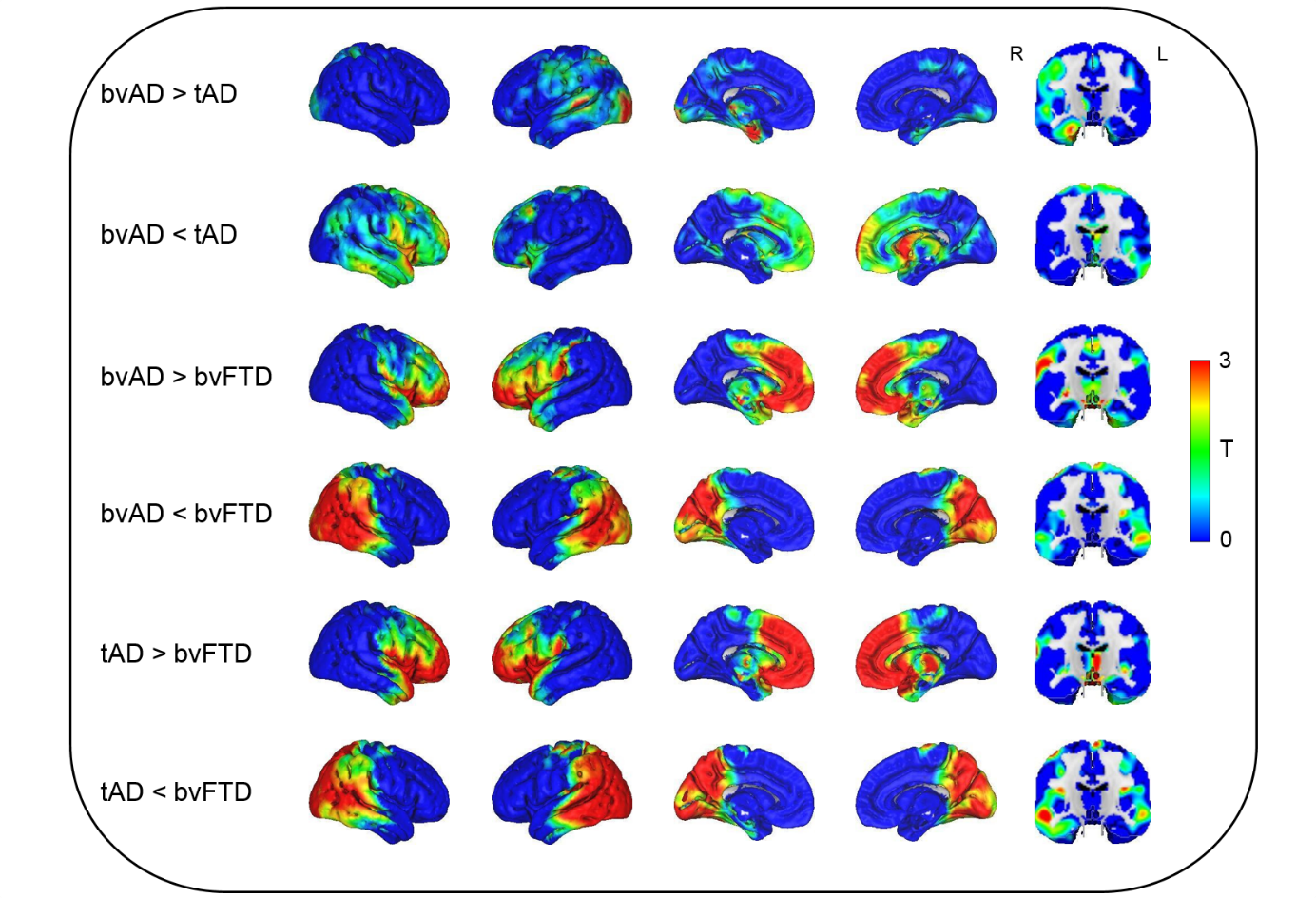
Patterns of based on t-maps.**

Surface rendering of *T*-maps resulting from showing hypometabolic regions in patients compared to other patient groups. Contrasts were adjusted for age and sex.

**
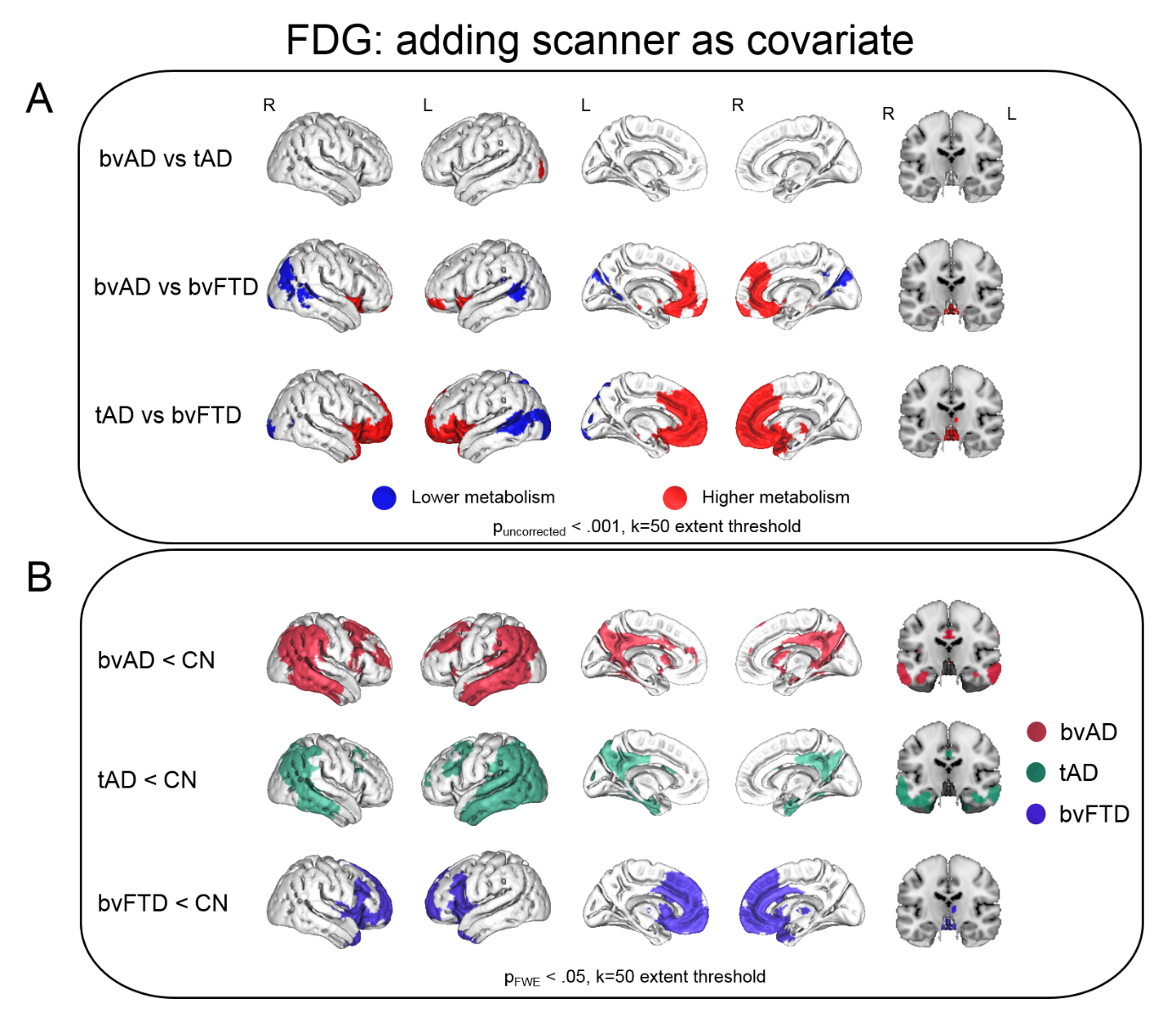
Patterns after adding scanner as covariate.**

*Panel A)* shows regions with significantly more or less metabolism in patient vs patient contrasts at P_uncorrected_ < 0.001, k=50 extent threshold. *Panel B)* shows regions showing less gray matter volume in patients vs cognitively normal controls at P_FWE_ < 0.05, k=50 extent threshold. Contrasts were adjusted for age, sex, and scanner type.
