# Supplementary material for: Investigating the clinico-anatomical dissociation in the behavioral variant of Alzheimer disease
Source: Alzheimers Res Ther. 2020 Nov 14;12:148. doi: 10.1186/s13195-020-00717-z (PMC7666520; doi:10.1186/s13195-020-00717-z)
Supplement: Supplementary file 8 — Additional file 8: : Supplement 8. Grey matter atrophy. [file 13195_2020_717_MOESM8_ESM.docx]

**Supplement 8 - Patterns of gray matter atrophy across patient groups.**

**
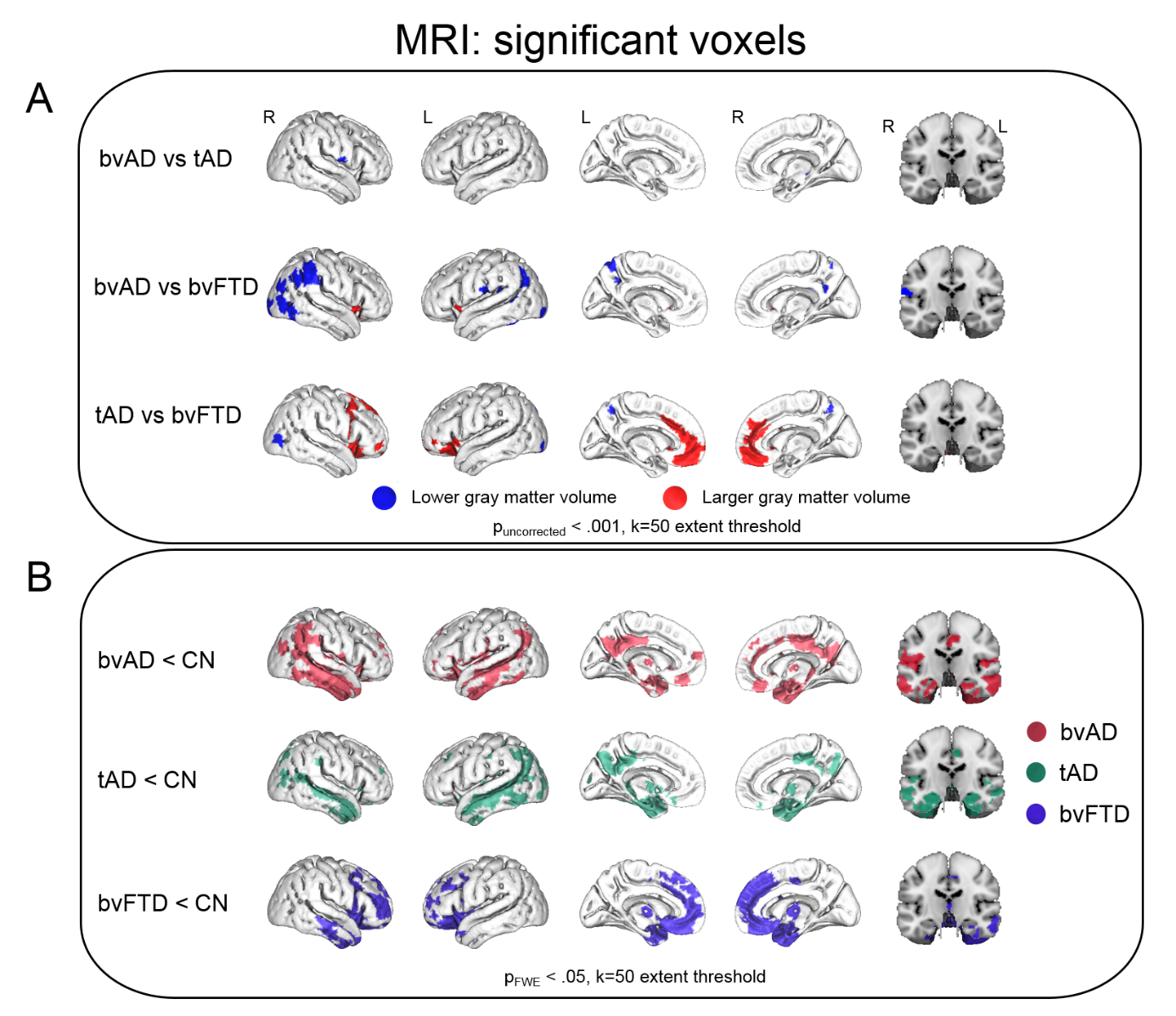
Patterns based on significant voxels.**

*Panel A)* shows regions with significantly more or less gray matter volume in patient vs patient contrasts at P_uncorrected_ < .001, k=50 extent threshold. *Panel B)* shows regions showing less gray matter volume in patients vs cognitively normal controls at P_FWE_ < .05, k=50 extent threshold. Contrasts were adjusted for age, sex, total intracranial volume and scanner field strength.

**
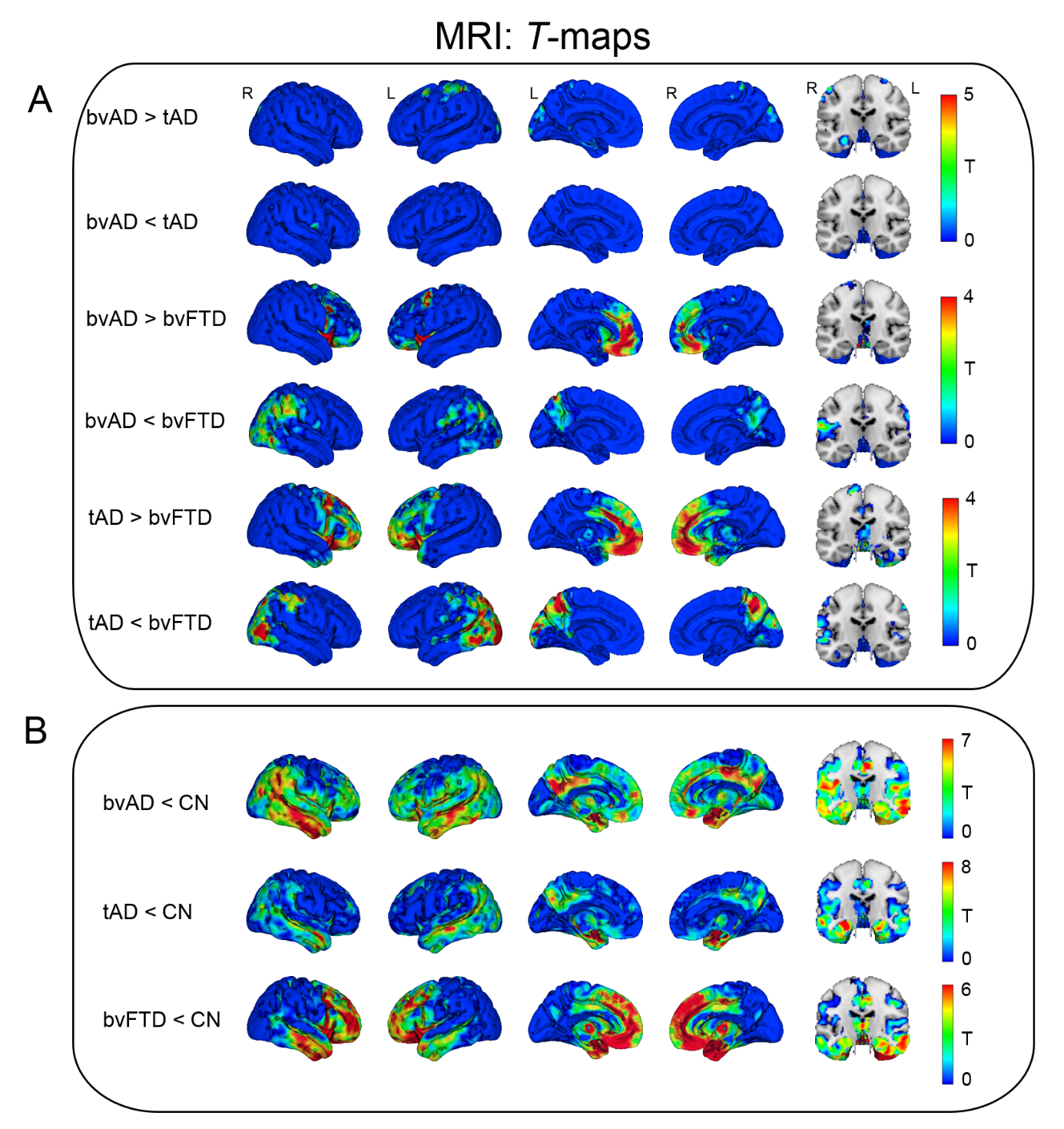
Patterns based on *T*-maps.**

Surface rendering of *T*-maps resulting from showing differences in gray matter volume regions. Contrasts were adjusted for age, sex, total intracranial volume and scanner field strength.
